# Psychological Distress, Quality of Life, and Nicotine Dependence Among Cigarette Smokers, E-Cigarette Users, and Non-Smokers in Jeddah, Saudi Arabia: A Cross-Sectional Study

**DOI:** 10.7759/cureus.115272

**Published:** 2026-08-26

**Authors:** Farah A Yahya, Reem F Alnemari, Asma Alahmari, Saad A Alghamdi

**Affiliations:** 1 Preventive Medicine, King Saud bin Abdul-Aziz University for Health Sciences, Jeddah, SAU; 2 Preventive Medicine, Armed Forces Hospital, Taif, SAU; 3 Preventive Medicine, Ministry of National Guard Health Affairs, Jeddah, SAU; 4 Preventive Medicine, King Abdullah International Medical Research Center, Jeddah, SAU; 5 Preventive Medicine, King Abdulaziz Medical City, Jeddah, SAU

**Keywords:** e-cigarettes, nicotine dependence, psychological distress, quality of life, saudi arabia, tobacco use

## Abstract

Background

Tobacco use continues to be a significant global public health issue, causing over 8 million deaths each year. People with mental health conditions face a higher risk, and new nicotine products have increased the complexity of the challenge. Despite this, there is limited information on psychological distress, nicotine dependence, and quality of life among cigarette and e-cigarette users.

Objective

The primary objective of this study was to compare psychological distress and quality of life among cigarette smokers, e-cigarette users, and non-smokers in Jeddah, Saudi Arabia. The secondary objective was to examine the association between nicotine dependence severity and psychological distress and quality of life among cigarette smokers and e-cigarette users.

Methods

A cross-sectional study was conducted among 368 adults (≥18 years) living in Jeddah from April to June 2026. Participants were recruited using stratified quota convenience sampling through online distribution via Telegram and WhatsApp applications, and community networks. The participants were divided into three groups: cigarette smokers (n=117), e-cigarette users (n=95), and non-smokers (n=156). Data were collected using a self-administered questionnaire covering sociodemographics, smoking history, psychological distress (K6), quality of life (World Health Organization Quality of Life - Brief Version (WHOQOL-BREF)), and nicotine dependence (Fagerström Test for Nicotine Dependence (FTND)/electronic Fagerström Test for Cigarette Dependence (e-FTCD)).

Results

Non-smokers scored higher than both smoking groups on most WHOQOL-BREF domains (p < 0.05). The mean K6 score was 15.20 (SD = 5.67), and 64.9% of participants screened positive for serious psychological distress. The proportion of serious psychological distress did not differ significantly by smoking status (p = 0.111). Among cigarette smokers and e-cigarette users, nicotine dependence severity showed a significant non-linear association with psychological distress (p = 0.001 and p = 0.004, respectively).

Conclusion

Non-smokers had higher WHOQOL-BREF scores than cigarette smokers and e-cigarette users on most domains. Although 64.9% of participants screened positive for serious psychological distress, this finding should be interpreted cautiously given the convenience sampling approach and may not be generalizable to the broader population. Further research is needed to clarify the relationship between nicotine dependence, psychological distress, and quality of life and to assess the potential implications for tobacco cessation and mental health interventions in Saudi Arabia.

## Introduction

Tobacco use remains a major global public health challenge, responsible for more than 8 million deaths annually and affecting over 1.3 billion individuals worldwide [1]. Despite substantial declines in combustible cigarette smoking in many high-income countries over the past two decades, progress has been uneven, and the emergence of novel nicotine products has added complexity to the overall risk profile rather than fully displacing harm [1,2]. Individuals with mental health conditions bear a disproportionate share of this burden, exhibiting higher smoking prevalence, accounting for a substantial proportion of tobacco-attributable mortality, and experiencing significantly reduced life expectancy compared with the general population [3].

Concurrently, the nicotine product landscape has diversified. While conventional cigarette use has plateaued or declined in several settings, e-cigarette use has increased among both smokers and non-smokers, raising concerns regarding whether these products serve primarily as harm-reduction alternatives or as additional drivers of nicotine dependence, particularly among psychologically vulnerable populations [2]. Surveillance data suggest that newer nicotine products may be contributing to a renewed wave of nicotine addiction, especially among young adults, with uncertain implications for long-term population health outcomes [1,2].

Nicotine dependence is commonly operationalized using validated behavioral instruments. The Fagerström Test for Nicotine Dependence (FTND) is among the most widely used tools in both clinical and epidemiologic research, quantifying dependence severity through indicators such as time to first cigarette and daily consumption [4]. Higher FTND scores are consistently associated with greater physiological dependence, lower cessation success, and poorer health outcomes [4]. When evaluated alongside multidimensional quality-of-life measures such as the World Health Organization Quality of Life - Brief Version (WHOQOL-BREF), greater nicotine dependence has been linked to significantly lower quality-of-life scores, suggesting a dose-response relationship between dependence severity and perceived well-being [5]. The combined use of the Kessler Psychological Distress Scale (K6), WHOQOL-BREF, and FTND within a single analytic framework enables a more integrated assessment of psychological distress, nicotine dependence, and quality-of-life outcomes [4,6,7].

Within high-risk populations, serious psychological distress (SPD) has emerged as a practical indicator of clinically relevant mental health morbidity in population-based research. SPD encompasses symptoms such as nervousness, hopelessness, restlessness, depressed mood, perceived effortfulness, and feelings of worthlessness and is operationalized using the 6-item Kessler Psychological Distress Scale (K6) [3,7]. Evidence from large national datasets indicates that adults with elevated psychological distress are more likely to be current smokers, consume cigarettes more heavily, and experience lower cessation success despite comparable quit intentions [3]. Population-based survey data demonstrate a persistently higher prevalence of cigarette use, e-cigarette use, and dual use among adults with SPD compared with those without distress [2,3]. These findings are consistent with broader syntheses identifying tobacco use among individuals with mental illness as a major public health challenge characterized by persistent dependence and excess mortality [2,3].

Quality of life has increasingly been recognized as a critical outcome when evaluating the downstream impact of nicotine use and dependence [5]. The World Health Organization Quality of Life-BREF (WHOQOL-BREF) is a widely used, cross-culturally validated instrument that assesses physical, psychological, social, and environmental domains, as well as overall quality of life and general health. The tool demonstrates strong psychometric performance and is particularly suitable for large-scale epidemiologic studies where minimizing respondent burden is essential [6]. In tobacco research, WHOQOL-BREF has consistently shown lower domain scores among current users, with further deterioration observed as dependence severity increases [5].

Saudi Arabia represents a relevant context for examining these relationships. A community-based cross-sectional study among young adults in Riyadh reported a current e-cigarette use prevalence of 10.5%, with higher use observed among males and individuals perceiving e-cigarettes as safer than conventional cigarettes [8]. A recent systematic review synthesizing 15 Saudi studies reported a median e-cigarette prevalence of 36.6%, with estimates ranging from approximately 7% among university students to nearly 80% among young adult samples [9]. Despite this growing literature, most Saudi studies have emphasized prevalence and attitudes, with limited integration of validated measures of psychological distress, nicotine dependence, and multidimensional quality of life within a single analytic model.

Collectively, existing evidence indicates that tobacco and nicotine products remain a substantial population health burden, that individuals with elevated psychological distress experience higher levels of cigarette and e-cigarette use, and that nicotine dependence is associated with measurable decrements in quality of life [2-5]. However, data examining the intersection of psychological distress, nicotine dependence, and quality of life among adult users of cigarettes and e-cigarettes in Saudi Arabia remain limited. This study aims to compare psychological distress and quality of life among cigarette smokers, e-cigarette users, and non-smokers in the Saudi population living in Jeddah in 2026.

## Materials and methods

Study design and setting

This study has a cross-sectional design aimed at assessing psychological distress and quality of life in three adult population groups: cigarette smokers, e-cigarette users, and non-smokers. Participants were recruited using stratified quota convenience sampling. Recruitment was conducted online through Telegram and WhatsApp groups and community networks targeting adults residing in Jeddah. Recruitment continued until the predefined quota for each study group was reached. An online eligibility screening process assessed age, Jeddah residency, and smoking status. Eligible participants who provided informed consent proceeded to the self-administered questionnaire.

Population

Eligible participants were adults aged 18 years or older who resided in Jeddah, Saudi Arabia, and provided electronic informed consent before completing the survey. Participants were classified into one of three mutually exclusive groups according to their smoking status. Cigarette smokers were defined as adults who had smoked at least 100 cigarettes during their lifetime and who currently smoked every day. E-cigarette users were defined as adults who currently used e-cigarettes every day and had a history of e-cigarette use. Non-smokers were defined as adults who had never used conventional cigarettes.

Individuals who reported concurrent use of both conventional cigarettes and e-cigarettes (dual users) were excluded to avoid potential confounding related to mixed nicotine exposure. Participants with self-reported psychiatric illness or multiple chronic comorbidities that could substantially affect quality of life were also excluded. 

Sample size

The sample size was calculated using G*Power 3.1 (developed by researchers at Heinrich Heine University Düsseldorf), as one-way analysis of variance (ANOVA) was planned to compare quality of life and psychological distress among cigarette smokers, e-cigarette users, and non-smokers. Assuming a medium effect size (Cohen's f = 0.25), a two-sided significance level of 0.05, and 80% power, a minimum of 52 participants per group was required (smokers = 52, e-cigarette users = 52, non-smokers = 52), giving a minimum total of 156 participants. The final number of participants who completed the survey was 368, exceeding the minimum required sample size.

Data collection tool

Data was collected using a structured, self-administered online questionnaire developed in Google Forms and provided in both Arabic and English. The questionnaire comprised six sections covering eligibility and electronic informed consent, sociodemographic characteristics, smoking history, psychological distress, quality of life, and nicotine dependence. Psychological distress was assessed using the Arabic-validated Kessler Psychological Distress Scale (K6) [7]. The six-item instrument yields a total score ranging from 0-24, with scores ≥13 indicating serious psychological distress (SPD). Four supplementary K6 items assessing the frequency and impact of distress were also included.

Quality of life was assessed using the Arabic version of the WHOQOL-BREF [6]. The instrument consists of 26 items covering the physical, psychological, social, and environmental domains, together with two items assessing overall quality of life and general health. Domain scores were transformed to a 0-100 scale according to WHO guidelines, with higher scores indicating better quality of life.

Psychological distress was assessed using the Kessler Psychological Distress Scale (K6) (Copyright © Ronald C. Kessler, PhD) [7]. The Arabic version was used in accordance with the developer’s terms for unrestricted use. Nicotine dependence was assessed using the Fagerström Test for Nicotine Dependence (FTND) for cigarette smokers and the E-cigarette Fagerström Test of Cigarette Dependence (e-FTCD) for e-cigarette users [10,11]. Both instruments generate total scores ranging from 0 to 10, with higher scores indicating greater nicotine dependence. Both instruments were translated into Arabic using forward translation. Face validity was assessed through pilot testing with five participants, resulting in minor wording modifications. Formal psychometric validation was not performed because of the limited pilot sample size.

Data analysis

Data was analyzed using IBM SPSS Statistics, version 26.0 (IBM Corp., Armonk, NY). Descriptive statistics were calculated for all sociodemographic variables and study outcomes. Categorical variables were presented as frequencies and percentages. Continuous variables were presented as means with standard deviations (SD) and medians with interquartile ranges (IQR) after assessing normality using the Shapiro-Wilk test and visual inspection of histograms and Q-Q plots. Differences in psychological distress (K6 scores) and quality of life (WHOQOL-BREF domain scores) across the three groups (cigarette smokers, e-cigarette users, and non-smokers) were compared. Given the non-normal distribution of the data, the Kruskal-Wallis test was used to compare continuous variables across the three groups. For categorical variables, differences across the three groups were assessed using the Chi-square test. The association between nicotine dependence severity (FTND and e-FTCD scores) and both psychological distress and quality-of-life outcomes was compared using the Kruskal-Wallis test for continuous variables and chi-square or Fisher's exact test for categorical variables. Statistical significance was set at p < 0.05 for all analyses, and all tests were two-tailed.

Ethical considerations

This study was conducted in accordance with the ethical principles for medical research involving human participants described in the World Medical Association Declaration of Helsinki (2024 revision) [12]. Participation was voluntary and based on electronic informed consent obtained prior to survey initiation.

## Results

A total of 368 participants completed the survey. As shown in Table 1, the majority were male (55.4%), with age distribution concentrated in the 25-44 age range (59.8%). Most participants held a university degree or higher (56.1%), were employed (66.7%), and had a monthly income of less than 10,000 SAR (67.5%). Regarding smoking, 31.8% (n=117) were cigarette smokers, 25.8% (n=95) were e-cigarette users, while most (42.4%, n=156) were non-smokers.

**Table 1:** Sociodemographic characteristics of the study population (N = 368)

Variable	Category	Total (N = 368)	Cigarette (N=117)	E-cigarette (N=95)	Non-smoker (N=156)
Gender, N (%)	Male	204 (55.4%)	79 (67.5%)	47 (49.5%)	78 (50.0%)
	Female	164 (44.6%)	38 (32.5%)	48 (50.5%)	78 (50.0%)
Age (years), N (%)	18-24	74 (20.1%)	14 (12%)	26 (27.4%)	34 (21.8%)
	25-34	113 (30.7%)	31 (26.5%)	28 (29.5%)	54 (34.6%)
	35-44	107 (29.1%)	42 (35.9%)	24 (25.3%)	41 (26.3%)
	45-54	38 (10.3%)	13 (11.1%)	11 (11.6%)	14 (9.0%)
	55-65	35 (9.5%)	17 (14.5%)	5 (5.3%)	13 (8.3%)
	>65	1 (0.3%)	0 (0%)	1 (1.1%)	0 (0%)
Educational level, N (%) (N=360)	Primary	7 (1.9%)	0 (0%)	3 (3.2%)	4 (2.6%)
	Preparatory	45 (12.5%)	18 (16.1%)	6 (6.5%)	21 (13.5%)
	Secondary	106 (29.4%)	34 (30.4%)	45 (48.4%)	27 (17.4%)
	University graduate or higher	202 (56.1%)	60 (53.6%)	39 (41.9%)	103 (66.5%)
Marital status, N (%) (N=363)	Single	147 (40.5%)	46 (40.0%)	34 (36.6%)	67 (43.2%)
	Married	134 (36.9%)	44 (38.3%)	32 (34.4%)	58 (37.4%)
	Divorced/Widow/prefer not to say	82 (22.6%)	25 (21.7%)	27 (29.0%)	30 (19.4%)
Monthly income (SAR), N (%) (N=326)	< 4,000	137 (42.0%)	29 (26.9%)	49 (55.7%)	59 (45.4%)
	4,000-10,000	83 (25.5%)	46 (42.6%)	8 (9.1%)	29 (22.3%)
	10,000-15,000	54 (16.6%)	15 (13.9%)	18 (20.5%)	21 (16.2%)
	> 15,000	52 (16.0%)	18 (16.7%)	13 (14.8%)	21 (16.2%)
Employment status, N (%) (N=351)	Employed	234 (66.7%)	87 (77.0%)	51 (60.0%)	96 (62.7%)
	Not Employed	117 (33.3%)	26 (23.0%)	34 (40.0%)	57 (37.3%)
Percentages are calculated based on available responses.					

**Figure 1:**
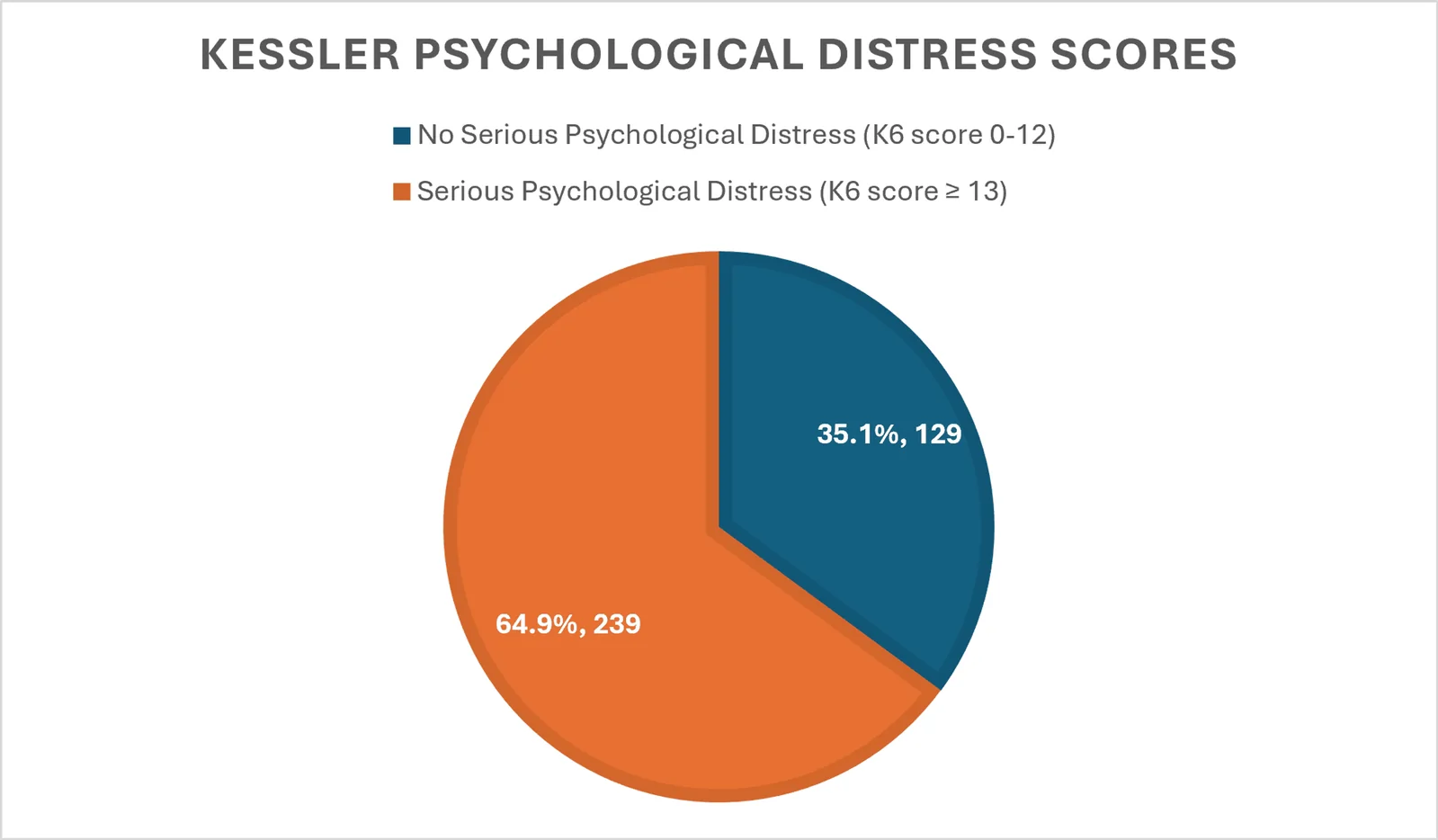
Kessler psychological distress scores among the study population (K6) (N=368)

Among the 212 participants who had ever smoked, the mean age of smoking initiation was 21.05 years (SD = 6.72), and the mean duration of smoking was 7.11 years (SD = 4.71). Approximately two-thirds (63.9%) reported smoking with friends, and nearly half (48.0%) indicated that all of their close friends smoked. As shown in Figure 1, the mean K6 total score was 15.20 (SD = 5.67), with a median of 15.50 (IQR = 10) (Range 0-24). Most participants (64.9%, n=239) met the criteria for serious psychological distress (K6 score ≥ 13), while 35.1% (n=129) had no serious psychological distress.

Table 2 presents a comparison of sociodemographic characteristics across the three smoking-status groups. Significant differences were observed for gender (p = 0.006), age (p = 0.036), educational level (p < 0.001), monthly income (p < 0.001), and employment status (p = 0.017).

**Table 2:** Sociodemographic characteristics by smoking status (N=368) *Chi-square test was used; χ² values are reported alongside p-values. Percentages are calculated based on available responses.

Variable	Category	Cigarette (N=117)	E-cigarette (N=95)	Non-smoker (N=156)	χ² (df)	p-value*
Gender, N (%)	Male	79 (38.7%)	47 (23.0%)	78 (38.2%)	χ²(2) = 10.150	0.006
	Female	38 (23.2%)	48 (29.3%)	78 (47.6%)		
Age (years), N (%)	18-24	14 (18.9%)	26 (35.1%)	34 (45.9%)	χ²(6) = 13.455	0.036
	25-34	31 (27.4%)	28 (24.8%)	54 (47.8%)		
	35-44	42 (39.3%)	24 (22.4%)	41 (38.3%)		
	45 and above	30 (40.5%)	17 (23.0%)	27 (36.5%)		
Educational level, N (%)	Primary/ Preparatory	18 (34.6%)	9 (17.3%)	25 (48.1%)	χ²(4) = 27.363	<0.001
	Secondary	34 (32.1%)	45 (42.5%)	27 (25.5%)		
	University graduate or higher	60 (29.7%)	39 (19.3%)	103 (51.0%)		
Marital status, N (%)	Single	46 (31.3%)	34 (23.1%)	67 (45.6%)	χ²(4) = 3.337	0.503
	Married	44 (32.8%)	32 (23.9%)	58 (43.3%)		
	Divorced/Widow/prefer not to say	25 (30.5%)	27 (32.9%)	30 (36.6%)		
Monthly income (SAR), N (%)	Less than 4,000	29 (21.2%)	49 (35.8%)	59 (43.1%)	χ²(6) = 33.790	<0.001
	4,000-10,000	46 (55.4%)	8 (9.6%)	29 (34.9%)		
	10,000-15,000	15 (27.8%)	18 (33.3%)	21 (38.9%)		
	Higher than 15,000	18 (34.6%)	13 (25.0%)	21 (40.4%)		
Employment status, N (%)	Employed	87 (37.2%)	51 (21.8%)	96 (41.0%)	χ²(2) = 8.179	0.017
	Not Employed	26 (22.2%)	34 (29.1%)	57 (48.7%)		

Males were more likely to be cigarette smokers (38.7% of males), while females were more likely to be e-cigarette smokers (29.3% of females) or non-smokers (47.6% of females). Younger participants (18-24 years) were more likely to use e-cigarettes (35.1%), whereas older participants (45+ years) were more likely to smoke cigarettes (40.5%). Participants with university-level education were predominantly non-smokers (51.0%), followed by cigarette smokers (29.7%), while those with secondary education were more likely to use e-cigarettes (42.5%). By income, e-cigarette users were more common in the lowest income bracket (<4,000 SAR; 35.8%), while cigarette smokers were most common in the middle-income bracket (4,000-10,000 SAR; 55.4%). Employed individuals were more likely to be cigarette smokers (37.2%), while unemployed individuals were more likely to be non-smokers (48.7%) or e-cigarette users (29.1%). No significant difference was found for marital status (p = 0.503).

Table 3 presents the comparison of quality-of-life domain scores across the three groups. Statistically significant differences were observed across all domains and overall perceptions (p < 0.05). Non-smokers consistently reported the highest scores across all domains. However, in the social relationship domain, cigarette smokers and non-smokers reported similar median scores (66.67 for both), while e-cigarette users reported the lowest score (58.33). Cigarette smokers reported lower scores than non-smokers in the physical (60.71 vs 75.00), psychological (58.33 vs 66.67), and environmental (65.63 vs 76.56) domains. E-cigarette users reported the lowest scores in the social (58.33) and environmental (56.25) domains.

**Table 3:** Comparison of quality-of-life domains by smoking status (WHOQOL-BREF) (N=368) *Kruskal-Wallis test was used; H-statistics with degrees of freedom are reported. Domain scores are transformed to a 0-100 scale according to WHO guidelines, with higher scores indicating better quality of life. WHOQOL-BREF: World Health Organization Quality of Life - Brief Version Domain scores are transformed to a 0–100 scale according to WHO guidelines, with higher scores indicating better quality of life.

Variable	Cigarette, median (IQR) (N=117)	E-cigarette, median (IQR) (N=95)	Non-smoker, median (IQR) (N=156)	H2 (df)	P-value*
Physical domain	60.71 (25)	67.86 (17.86)	75 (17.86)	13.970	0.001
Psychological domain	58.33 (29.17)	58.33 (12.50)	66.67 (16.67)	25.340	<0.001
Social relationship domain	66.67 (41.67)	58.33 (33.33)	66.67 (33.33)	13.783	0.001
Environment domain	65.63 (28.13)	56.25 (15.63)	76.56 (21.88)	37.361	<0.001
The overall perception of QoL	3 (1)	3 (1)	4 (1)	36.373	<0.001
The overall perception of health	3 (2)	4 (1)	4 (2)	21.982	<0.001

Table 4 presents the comparison of serious psychological distress by smoking status. The proportion of participants screening positive for serious psychological distress was highest among non-smokers (69.9%), followed by cigarette smokers (65.0%) and e-cigarette users (56.8%). However, the difference in the prevalence of serious psychological distress across the three groups was not statistically significant (χ²(2) = 4.403, p = 0.111).

**Table 4:** Comparison of Kessler psychological distress by smoking status (K6) (N=368) *Chi-square test was used; χ² value with degrees of freedom is reported.

Variable	Cigarette (N=117)	E-cigarette (N=95)	Non-smoker (N=156)	χ² (df)	P-value*
No Serious Psychological Distress (K6 score 0-12), N (%)	41 (31.8%)	41 (31.8%)	47 (36.4%)	χ²(2) = 4.403	0.111
Serious Psychological Distress (K6 score ≥ 13), N (%)	76 (31.8%)	54 (22.6%)	109 (45.6%)		

**Figure 2:**
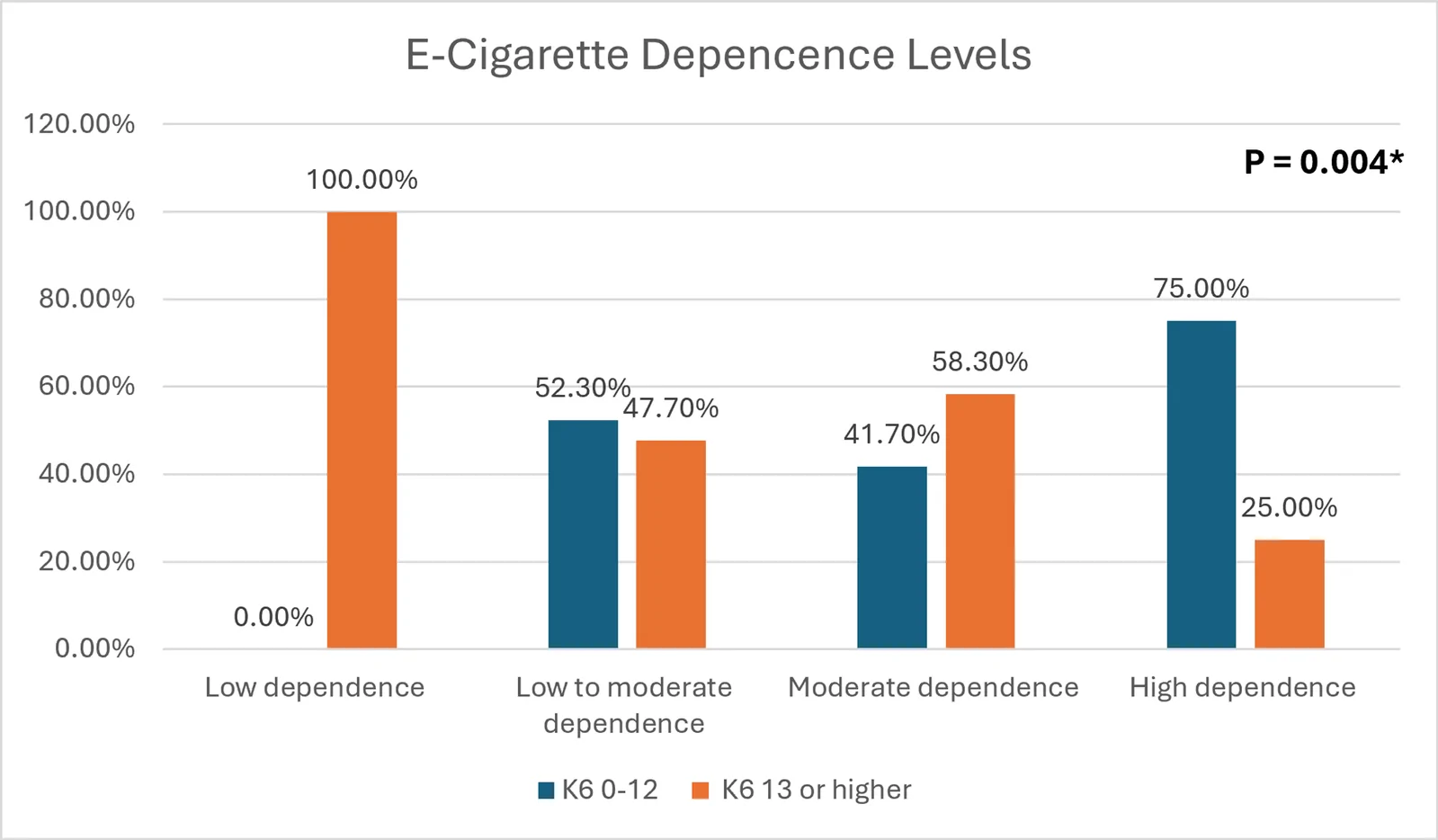
Association of e-cigarette dependence levels and psychological distress (e-FTCD) (N=95) *Fisher’s Exact test was used. e-FTCD: e-cigarette Fagerström Test of Cigarette Dependence.

Figure 2 presents the association between e-cigarette dependence levels (e-FTCD) and Kessler psychological distress categories (K6). Among participants with low dependence, all (100.0%) had serious psychological distress (K6 score ≥ 13). Among those with low to moderate dependence, 52.3% had no serious psychological distress, while 47.7% had serious psychological distress (K6 score ≥ 13). Among participants with moderate dependence, 41.7% had no serious psychological distress, while 58.3% had serious psychological distress. Among those with high dependence, 75.0% had no serious psychological distress, while 25.0% had serious psychological distress. The association between e-cigarette dependence level and psychological distress was statistically significant (p = 0.004).

**Figure 3:**
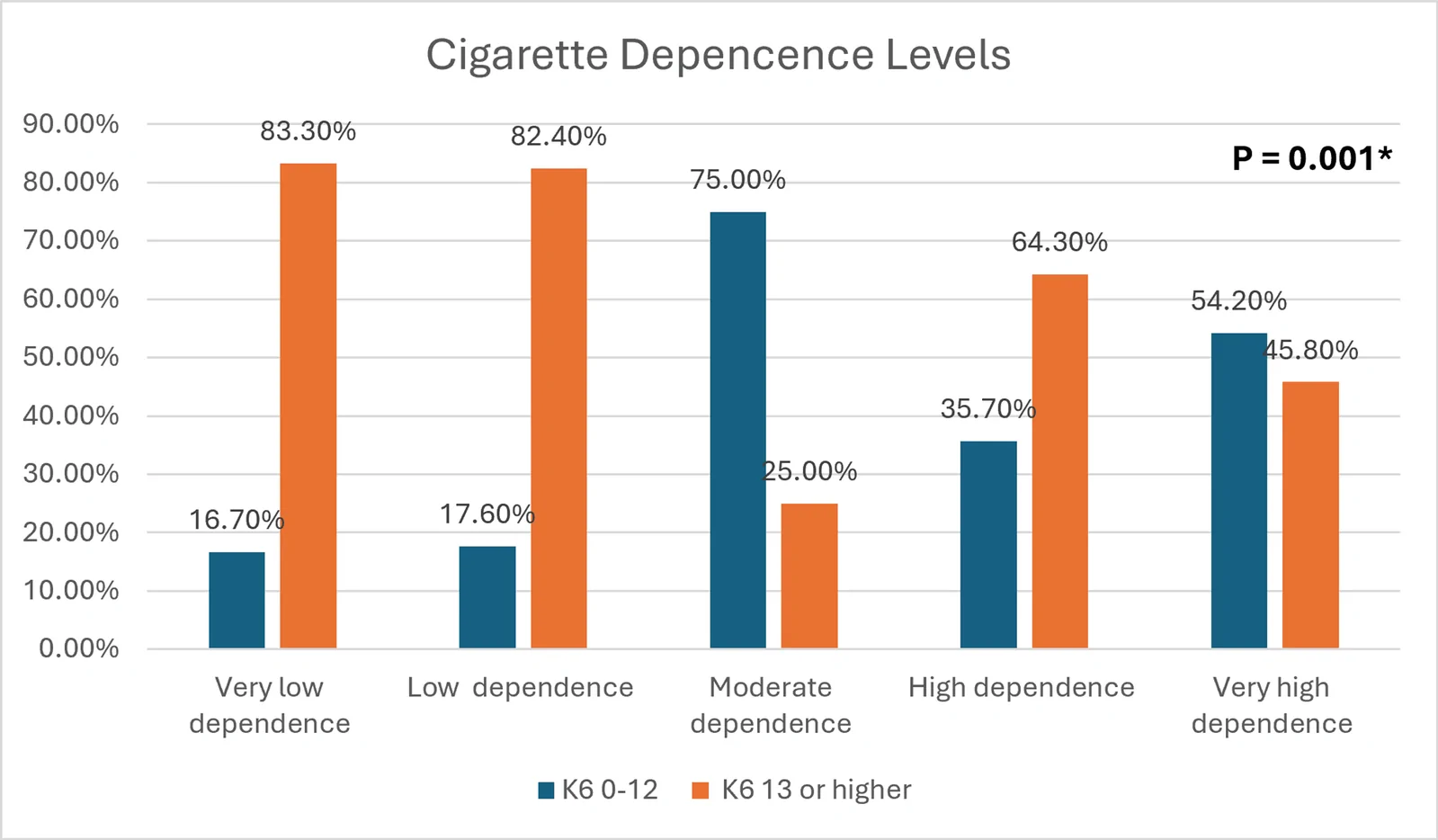
Association of cigarette dependence levels and psychological distress (FTND) (N=117) *Fisher's exact test was used.

Figure 3 presents the association between cigarette dependence levels (FTND) and psychological distress categories (K6). Among participants with very low dependence, 16.7% had no serious psychological distress, while 83.3% had serious psychological distress (K6 score ≥ 13). Among those with low dependence, 17.6% had no serious psychological distress, while 82.4% had serious psychological distress. Among participants with moderate dependence, 75.0% had no serious psychological distress, while 25.0% had serious psychological distress. Among those with high dependence, 35.7% had no serious psychological distress, while 64.3% had serious psychological distress. Among participants with very high dependence, 54.2% had no serious psychological distress, while 45.8% had serious psychological distress. The association between cigarette dependence level and psychological distress was statistically significant (p = 0.001).

Table 5 presents the comparison of quality-of-life domains across e-cigarette dependence levels. Statistically significant differences were observed for the physical domain (p = 0.018), psychological domain (p = 0.005), social relationship domain (p = 0.019), environmental domain (p < 0.001), and overall perception of quality of life (p = 0.002). No significant difference was found for overall perception of health (p = 0.105). Higher dependence levels were generally associated with lower quality-of-life scores, although this pattern was not strictly linear across all domains. The lowest scores were consistently observed in the high-dependence group for the psychological (median = 41.67), social (median = 33.33), and overall health (median = 3) domains.

**Table 5:** Comparison of quality-of-life domains by e-cigarette dependence levels (e-FTCD) (N=95) *Kruskal-Wallis test was used; H-statistics with degrees of freedom are reported.

Domains	Low, median (IQR)	Low to moderate, median (IQR)	Moderate, median (IQR)	High, median (IQR)	H3 (df)	P- value*
Physical domain	78.57 (28.57)	67.86 (9.8)	60.71 (25)	71.43 (8.04)	10.018	0.018
Psychological domain	70.83 (25)	58.33 (12.50)	54.17 (12.50)	41.67 (25)	13.063	0.005
Social relationship domain	66.67 (33.33)	50 (39.58)	66.67 (25)	33.33 (31.25)	9.941	0.019
Environment domain	75 (21.88)	62.50 (17.97)	53.13 (7.81)	56.25 (16.41)	18.364	<0.001
The overall perception of QoL	4 (1)	3 (2)	4 (1)	3 (2)	14.563	0.002
The overall perception of health	4 (0)	4 (1)	4 (0)	3 (2)	6.132	0.105

Table 6 presents a comparison of quality-of-life domains across levels of cigarette dependence. Statistically significant differences were observed for the physical domain (p = 0.013), social relationship domain (p = 0.004), the environment domain (p = 0.002), and overall perception of health (p = 0.001). No significant differences were found in the psychological domain (p = 0.261) or in overall perception of quality of life (p = 0.142). Participants with very low dependence reported the highest scores for social relationships (median = 83.33), environmental domain (median = 75.00), and overall health (median = 4.00). Those with moderate, high, or very high dependence generally reported lower scores than those with very low dependence, although the very high dependence group showed somewhat higher scores in the social and environmental domains compared to the moderate and high dependence groups.

**Table 6:** Comparison of quality-of-life domains by cigarette dependence levels (FTND) (N=117) *Kruskal-Wallis test was used; H-statistics with degrees of freedom are reported.

Domains	Very low, median (IQR)	Low, median (IQR)	Moderate, median (IQR)	High, median (IQR)	Very high, median (IQR)	H4 (df)	P- value*
Physical domain	71.43 (28.57)	64.29 (23.21)	55.36 (17.86)	60.71 (18.75)	48.21 (28.57)	12.750	0.013
Psychological domain	66.67 (20.83)	66.67 (25.00)	50.00 (26.04)	50.00 (37.50)	54.17 (29.17)	5.269	0.261
Social relationship domain	83.33 (25.00)	66.67 (58.33)	45.83 (41.67)	58.33 (41.67)	66.67 (29.17)	15.148	0.004
Environment domain	75.00 (31.25)	59.38 (34.38)	59.38 (14.06)	53.13 (21.09)	67.19 (30.47)	17.436	0.002
The overall perception of QoL	3.00 (1.00)	4.00 (2.00)	3.00 (0.00)	3.00 (1.00)	3.00 (2.00)	6.894	0.142
The overall perception of health	4.00 (1.00)	3.00 (2.00)	2.50 (2.00)	3.00 (1.00)	3.00 (2.00)	18.685	0.001

## Discussion

This study aimed to compare psychological distress and quality of life among cigarette smokers, e-cigarette users, and non-smokers in the Saudi population living in Jeddah during 2026, and to assess the association between nicotine dependence severity and both psychological distress and quality of life. The findings showed that non-smokers had higher WHOQOL-BREF scores than cigarette smokers and e-cigarette users on most domains. A high proportion of participants (64.9%) screened positive for serious psychological distress on the K6 scale. Moreover, nicotine dependence severity was significantly associated with psychological distress among both cigarette smokers and e-cigarette users, although the observed associations were non-linear. Higher nicotine dependence was also associated with lower scores across several WHOQOL-BREF domains, although these associations were not consistent across all domains. These results contribute to the limited evidence examining the intersection of nicotine use, mental health, and quality of life in Saudi Arabia.

Prevalence and patterns of tobacco and e-cigarette use

In our sample, the prevalence of current cigarette smoking was 31.8%, while e-cigarette use stood at 25.8%. These figures are broadly in line with recent national data. Albarrak and colleagues (2023), for instance, reported past-30-day tobacco use of 19.8% and e-cigarette use of 21.1% in their Saudi sample [13]. The somewhat higher estimates observed in our study may reflect differences in the study population or sampling methodology compared with previous national studies. A recent systematic review of 15 Saudi studies reported a median e-cigarette prevalence of 36.6%, with individual estimates ranging from about 7% among university students to nearly 80% in young adult samples [9]. Our finding of 25.8% falls within this reported range, reinforcing the view that e-cigarette use has become a significant public health issue in Saudi Arabia.

Quality of life and smoking status

One of the notable findings was that non-smokers generally reported higher quality-of-life scores than cigarette smokers and e-cigarette users across several WHOQOL-BREF domains. Significant differences across smoking-status groups were observed for physical health (p = 0.001), psychological health (p < 0.001), social relationships (p = 0.001), environmental health (p < 0.001), and both overall quality of life and general health perception (both p < 0.001). These findings are broadly consistent with previous literature reporting lower quality of life among tobacco users compared with non-smokers [5]. A prior Saudi study comparing cigarette, e-cigarette, and dual users also found substantial long-term declines in quality of life among users compared to never-users [14]. Other investigations have similarly reported that smokers tend to fare worse than non-smokers on physical, psychological, social, and environmental dimensions of quality of life [5].

Interestingly, e-cigarette users in our study scored lower than cigarette smokers in both the social relationships domain (median = 58.33 vs. 66.67) and the environmental domain (median = 56.25 vs. 65.63). This contrasts with some earlier reports suggesting that e-cigarette users may experience better health outcomes than cigarette smokers [14]. That said, other work has shown that exclusive e-cigarette use is associated with better self-rated health than exclusive cigarette smoking, particularly among moderate and heavy smokers [13]. The differences between our findings and those of previous studies may stem from variations in sample characteristics, differences in duration of use, or the possibility that many e-cigarette users in our sample were former smokers who switched to vaping, a transition that may not fully restore quality of life to the level of never-smokers. It is worth noting that a study of young adults found that exclusive e-cigarette users had the least impairment in lung function and quality of life when compared to cigarette-only and dual users [14].

Psychological distress and smoking status

The average K6 score in our sample was 15.20 (SD = 5.67), and 64.9% of participants screened positive for serious psychological distress using the K6 cutoff of ≥13. This proportion is higher than estimates reported in previous studies; for example, a Riyadh-based investigation of e-cigarette users reported that 42.2% had high distress levels [13]. The relatively high proportion observed in our study may be related, in part, to the sampling methodology. Participants were recruited through an online convenience survey, which may have preferentially attracted individuals experiencing psychological distress or other health concerns. Therefore, this prevalence should be interpreted cautiously and should not be considered representative of the broader population.

Contrary to our expectations, the comparison of psychological distress categories by smoking status did not show a statistically significant difference (p = 0.111). Although the proportion of participants screening positive for serious psychological distress was numerically highest among non-smokers (69.9%), followed by cigarette smokers (65.0%) and e-cigarette users (56.8%), this observed difference should not be interpreted as an association between non-smoking status and greater psychological distress. The higher observed proportion among non-smokers may reflect sampling variation or differences in sociodemographic and other participant characteristics across the groups. In particular, significant differences were observed across groups in age, sex, education, income, and employment status, and these factors were not adjusted for in the primary group comparisons. The finding therefore warrants cautious interpretation and further investigation.

Nicotine dependence and psychological distress

Nicotine dependence severity was significantly associated with psychological distress among both cigarette smokers (p = 0.001) and e-cigarette users (p = 0.004). This finding is broadly consistent with previous research from Taif City reporting an association between mental health status and nicotine dependence among adult cigarette smokers [15]. However, the observed patterns across dependence categories were non-linear and did not demonstrate a consistent directional relationship. Among cigarette smokers, the prevalence of serious psychological distress was highest among participants with very low (83.3%) and low (82.4%) dependence, decreased among those with moderate dependence (25.0%), and increased again among those with high dependence (64.3%), before decreasing among those with very high dependence (45.8%). Among e-cigarette users, serious psychological distress was observed in 100.0% of participants with low dependence, compared with 47.7% among those with low-to-moderate dependence, 58.3% among those with moderate dependence, and 25.0% among those with high dependence. These findings indicate a significant but non-linear association between nicotine dependence severity and psychological distress rather than a simple relationship in which greater dependence is associated with greater distress. The findings should be interpreted cautiously given the cross-sectional design, relatively small numbers within some dependence categories, and the unadjusted nature of the analysis. Further research using larger samples and adjusted analyses is needed to clarify this relationship.

Nicotine dependence and quality of life

Higher nicotine dependence severity was associated with lower scores across several WHOQOL-BREF domains, although the pattern was not consistent across all domains or between the two user groups. Among e-cigarette users (Table 5), significant differences were observed for physical (p = 0.018), psychological (p = 0.005), social (p = 0.019), environmental (p < 0.001), and overall quality of life (p = 0.002). The high-dependence group had the lowest scores for the psychological (median = 41.67) and social (median = 33.33) domains, suggesting that severe nicotine dependence is particularly detrimental to mental and social well-being. Among cigarette smokers (Table 6), significant differences were observed for physical (p = 0.013), social (p = 0.004), environmental (p = 0.002), and overall health perception (p = 0.001), whereas psychological health (p = 0.261) and overall quality of life (p = 0.142) did not differ significantly.

These findings are consistent with previous studies demonstrating an inverse relationship between nicotine dependence and quality of life [5]. The present study extends this evidence to a Saudi adult population and shows that the same relationship applies to both cigarette and e-cigarette users.

Sociodemographic correlates

Significant sociodemographic differences were observed across the three smoking status groups. Males were overrepresented among cigarette smokers (67.5%) compared to e‑cigarette users (49.5%) and non‑smokers (50.0%). This is in keeping with the well‑documented pattern of higher male smoking rates in Saudi Arabia and internationally [8,9,13]. Age distribution also differed significantly: e‑cigarette users were more concentrated in the younger age brackets (18-24 and 25-34 years), while cigarette smokers were more prevalent in the 35-44 year age group. This age gradient aligns with the view that e‑cigarettes are particularly popular among younger adults, who may view them as a less harmful alternative to conventional cigarettes [8,9].

Educational level showed a notable pattern: e‑cigarette users had the highest proportion of secondary education (48.4%), while non‑smokers had the highest proportion of university graduates (66.5%). This is consistent with Aljohani and colleagues (2024), who reported that many e‑cigarette users are well‑educated young adults [16]. Monthly income also differed significantly: e‑cigarette users were more likely to report lower incomes (< 4,000 SAR, 55.7%), whereas cigarette smokers were more likely to fall within the 4,000-10,000 SAR range (42.6%). Employment status revealed that cigarette smokers had the highest proportion of employed individuals (77.0%), while non‑smokers had the highest proportion of unemployed individuals (48.7%) [16]. Marital status was the only sociodemographic variable that did not differ significantly across groups (p = 0.503). These findings underscore the complex sociodemographic profile of tobacco and nicotine users in Saudi Arabia and highlight the need for public health interventions that are tailored to these demographic differences.

Strengths and limitations

This study has several strengths. It is among the first in Saudi Arabia to simultaneously examine psychological distress, quality of life, and nicotine dependence using the K6, WHOQOL-BREF, FTND, and e-FTCD within a single analytical framework [3,6,7,10,11]. The use of established instruments, together with Arabic translation and face-validity assessment where applicable, supports the standardized assessment of psychological distress, quality of life, and nicotine dependence. The target sample size of 368 participants was achieved.

At the same time, several limitations should be considered. The cross-sectional design precludes causal inference regarding the relationships between smoking, nicotine dependence, psychological distress, and quality of life [13,14]. Reliance on an online self-administered questionnaire may have introduced selection bias, and recruitment through convenience sampling limits the generalizability of the findings to the broader Saudi population. Furthermore, the primary comparisons between smoking-status groups were unadjusted for potentially important sociodemographic differences, including age, sex, education, income, and employment status; therefore, residual confounding cannot be excluded. In addition, dual users of cigarettes and e-cigarettes were excluded; therefore, the findings may not be generalizable to this growing population. Finally, smoking status and nicotine dependence were self-reported and may be subject to recall bias and social desirability bias.

## Conclusions

This study offers meaningful insights into the associations between smoking status, nicotine dependence, psychological distress, and quality of life among adults in Jeddah, Saudi Arabia. Non-smokers reported higher WHOQOL-BREF scores than cigarette smokers and e-cigarette users on most domains. Nicotine dependence severity was significantly associated with psychological distress among both cigarette smokers and e-cigarette users, with non-linear patterns observed across dependence categories, and was associated with lower scores across several WHOQOL-BREF domains. Further research using larger samples and adjusted analyses is needed to clarify the relationships between nicotine dependence, psychological distress, and quality of life and to inform future tobacco cessation and mental health interventions in Saudi Arabia.
